# Hybrid Materials Based on Multi-Walled Carbon Nanotubes and Nanoparticles with Antimicrobial Properties

**DOI:** 10.3390/nano11061415

**Published:** 2021-05-27

**Authors:** Madalina Elena David, Rodica-Mariana Ion, Ramona Marina Grigorescu, Lorena Iancu, Alina Maria Holban, Adrian Ionut Nicoara, Elvira Alexandrescu, Raluca Somoghi, Mihaela Ganciarov, Gabriel Vasilievici, Anca Irina Gheboianu

**Affiliations:** 1 National Institute for Research & Development in Chemistry and Petrochemistry - ICECHIM, 060021 Bucharest, Romania; rodica_ion2000@yahoo.co.uk (R.-M.I.); rmgrigorescu@gmail.com (R.M.G.); lorenna77ro@yahoo.com (L.I.); elvira.alexandrescu@icechim-pd.ro (E.A.); ralucasomoghi@outlook.com (R.S.); mihaela.ganciarov@icechim.ro (M.G.); gabi.vasilievici@gmail.com (G.V.); 2 Doctoral School of Materials Engineering Department, Valahia University of Targoviste, 130004 Targoviste, Romania; 3 Faculty of Biology, University of Bucharest, 060101 Bucharest, Romania; alina_m_h@yahoo.com; 4 Faculty of Applied Chemistry and Materials Science, University Politehnica of Bucharest, 011061 Bucharest, Romania; adi.nicoara18@gmail.com; 5 Institute of Multidisciplinary Research for Science and Technology, Valahia University of Targoviste, 130004 Targoviste, Romania; anca_b76@yahoo.com

**Keywords:** hybrid materials, decorated nanotubes, nanoparticles, antimicrobial properties, biofilm control

## Abstract

In this study, multi-walled carbon nanotubes (MWCNTs) were decorated with different types of nanoparticles (NPs) in order to obtain hybrid materials with improved antimicrobial activity. Structural and morphological analysis, such as Fourier transformed infrared spectroscopy, Raman spectroscopy, X-ray diffraction, transmission electron microscopy, environmental scanning electron microscopy/energy-dispersive X-ray spectroscopy and the Brunauer–Emmett–Teller technique were used in order to investigate the decoration of the nanotubes with NPs. Analysis of the decorated nanotubes showed a narrow size distribution of NPs, 7–13 nm for the nanotubes decorated with zinc oxide (ZnO) NPs, 15–33 nm for the nanotubes decorated with silver (Ag) NPs and 20–35 nm for the nanotubes decorated with hydroxyapatite (HAp) NPs, respectively. The dispersion in water of the obtained nanomaterials was improved for all the decorated MWCNTs, as revealed by the relative absorbance variation in time of the water-dispersed nanomaterials. The obtained nanomaterials showed a good antimicrobial activity; however, the presence of the NPs on the surface of MWCNTs improved the nanocomposites’ activity. The presence of ZnO and Ag nanoparticles enhanced the antimicrobial properties of the material, in clinically relevant microbial strains. Our data proves that such composite nanomaterials are efficient antimicrobial agents, suitable for the therapy of severe infection and biofilms.

## 1. Introduction

In the last decades, carbon nanotubes (CNTs) received increasing attention due to their unique characteristics (chemical, physical and mechanical properties), which make them suitable to be used in different areas, including industry, medicine, energy storage and environmental protection [1,2,3].

CNTs are allotropes of carbon consisting of a hexagonal arrangement of hybridized carbon atoms. They are assembled in cylindrical tubes and can be formed by a single sheet of graphene (single-walled carbon nanotubes, SWCNTs) or by multiple sheets of graphene linked through van der Waals non-covalent forces (multi-walled carbon nanotubes, MWCNTs) [4,5,6]. CNTs can be synthetized by several methods, such as arc-discharge, chemical vapor deposition, laser ablation, hydrothermal method and electrolysis. These methods present several disadvantages, like high costs, low purity or catalysts use. Instead, the chemical method seems to be a good option to obtain MWCNTs more easily, because it uses low temperature, it does not require the use of metal catalyst or pressures and is an inexpensively and environment-friendly technique [7]. The nanotubes’ structure depends closely on the CNTs synthesis method. Thus, the applications of these nanotubes are linked by their structure (such as: number of walls, length, diameter, structural quality, purity, etc.), which gives specific properties to CNTs [4]. Both types of CNTs have found applications in several fields, such as medicine (due to their optical properties [8]), solar thermal applications (favorable stability [9]), biosensing applications (electron transfer kinetics [10,11]), antimicrobial applications (strong antimicrobial activity against a variety of bacterial strains [12,13,14]) and drug delivery applications (due to their large surface/weight ratio [15,16]). Even so, MWCNTs present advantages compared with SWCNTs, such as easier mass production, low-cost synthesis and enhanced thermal and chemical stability [17].

With the rapid development of industries, more advanced materials are required. A solution regarding this problem was found in designing hybrid materials that will combine the properties of individual components. Surface modification of carbon nanotubes imparts new properties to these materials for various applications, where enhanced CNTs functionality, dispersion and compatibility are required. The most common methods to functionalize CNTs includes oxidation using various acids, when oxygen functional groups (–OH, –COOH) result. Oxygen-containing groups created on nanotubes’ surface promote the exfoliation of CNT bundles, enhance the solubility in polar media and offer the possibility of further modification [17].

MWCNTs decorated with nanoparticles (NPs) are a new family of innovative nanocomposites, effective for multiple biomedical applications [18]. It has been reported that antimicrobial activity of MWCNTs has significantly increased with the presence of NPs onto their surface [19]. In another study, it was reported that MWCNTs decorated with silver (Ag) NPs inactivated or killed the bacteria once with the increase of the exposure time [20]. Dinh and co-workers studied the antibacterial effect of MWCNTs, Ag NPs and MWCNTs_Ag nanocomposites against *Escherichia coli* and *Staphylococcus aureus* bacteria. It was reported that the nanocomposites (MWCNTs_Ag) show a larger inhibition zone, compared with Ag NPs and MWCNTs. These results suggest that the bacterial growth inhibition of the composite sample was a synergetic effect resulting from both the nanoparticles and MWCNTs. Additionally, the interaction and bactericidal mechanism of MWCNTs decorated with Ag NPs was reported to occur due to the physical interaction of the nanomaterials with cell membrane, the faster destructibility of cell membrane and disruption of membrane function, leading to cell death [21]. On the other hand, Mohan and co-workers investigated the antimicrobial action of hybrid nanocomposites based on MWCNTs and Ag NPs obtained via an environmentally friendly method. It has been reported that higher inhibitory effects were obtained against *E. coli*, compared with MWCNTs or Ag NPs, while in the case of *Pseudomonas aregunosa*, the MWCNTs decorated with Ag NPs were the least effective [22]. Additionally, a lesser inhibition zone of MWCNTs decorated with Ag NPs, compared with pure MWCNTs, was reported in other studies. It can be confirmed that in the case of the Gram-negative bacteria, MWCNTs are slightly more effective than CNTs decorated with Ag NPs, while for the Gram-positive bacteria, the decorated nanocomposites are more effective [23]. Sui and co-workers reported that MWCNTs decorated with zinc oxide (ZnO) NPs presented a powerful bactericidal effect against *E. coli* at a concentration of 0.5 mg/mL after 3 h, confirming that the combination of ZnO NPs and MWCNTs improved the antibacterial property of the nanocomposite [24]. In another study, the antimicrobial efficiency of raw MWCNTs, purified MWCNTs and MWCNTs decorated with ZnO against *E. coli* was investigated. It was reported that no obvious antimicrobial activity was observed against *E. coli* after being treated with raw MWCNTs (0.5 mg/mL), while for purified MWCNTs, the growth time of the cells was delayed by approximately 4 h, at the same concentration. In the case of MWCNTs coated with ZnO, there was no obvious cell growth observed within 20 h, at the same concentration [25]. Akhavan and co-workers reported that the antimicrobial activity of ZnO nanoparticles deposited on MWCNTs against *E. coli* increased with the gradual increase of the MWCNTs content [26]. The antimicrobial activity of MWCNTs decorated with hydroxyapatite (HAp) has been very little investigated, for example, Sivaraj and Vijayalakshmi studied the antimicrobial activity of *E. coli* and *S. aureus* on Ag-substituted HAp deposited on MWCNTs. It was reported that the higher inhibition zone was obtained at an increased concentration of Ag-HA into the walls of the carbon nanotubes, for both types of bacteria. In addition, it was reported that a higher zone of inhibition of *E. coli* could be attributed to less cell wall density compared with Gram-positive bacteria. The enhanced antimicrobial activity is related to the amount of Ag, mainly due to the variation in negative surface charge induced from the composite. The Ag ions released from the nanocomposite attack the cell wall membrane, penetrate into the cell and disrupt the DNA structure [27].

In this study, MWCNTs obtained by a new, inexpensive and simple technique, chemical synthesis, were used as supporting material for the nanoparticles. The major advantage of the chemical method refers to the fact that it does not require the use of metal catalyst, thus becoming an environment-friendly method. Furthermore, the obtained products were already functionalized with carboxylated groups from the synthesis procedure, so the decoration of the MWCNTs with nanoparticles was performed more easily [18].

The aim of this study was to design and characterize hybrid materials composed by MWCNTs, obtained by a new and environment-friendly technique, and different types of nanoparticles, in order to obtain efficient nanosystems, with low cost, fast to obtain and improved antimicrobial activity. In this study, synthetized MWCNTs were decorated with NPs, such as hydroxyapatite (MWCNTs_HAp), silver (MWCNTs_Ag) and zinc oxide (MWCNTs_ZnO), in order to obtain efficient hybrid materials based on MWCNTs. The obtained nanomaterials were tested against five microbial species, two Gram-positive (*S. aureus, Bacillus subtilis*), two Gram-negative (*Pseudomonas aeruginosa, E. coli*) and one yeast (*Candida albicans*) to cover the most important model opportunistic pathogens.

## 2. Materials and Methods

### 2.1. Materials and Synthesis

MWCNTs used in this study were obtained by chemical synthesis, purified [18] and then decorated with NPs. Additionally, the synthesis of MWCNTs_HAp was described and investigated in the same paper [18] and the obtained nanomaterials will be further used in order to examinate their antimicrobial activity. The in situ decoration of MWCNTs_ZnO and MWCNTs_Ag is presented below.

#### 2.1.1. In Situ Decoration of MWCNTs with ZnO Nanoparticles

The purified MWCNTs (5 wt%) were added to an aqueous solution of zinc nitrate (Zn(NO_3_)_2_·6H_2_O—5 wt%, Lach-ner, Neratovice, Cehia), and the resulting solution was sonicated for 2 h. Subsequently, 0.5 wt% citric acid (Sigma-Aldrich, Schnelldorf, Germany) and 5 wt% ethylene glycol (Sigma-Aldrich) solution was added in order to avoid the ZnO NPs aggregation, and to improve their dispersion in the environment. The mixture was stirred for another 2 h at room temperature. Then, ammonium hydroxide (NH_4_OH, Sigma-Aldrich) was slowly dropped into solution to a pH of 7, and the temperature was raised to 80 °C for 8 h. The material was calcined at 300 °C for 3 h in order to obtain MWCNTs decorated with zinc oxide (MWCNT_ZnO) NPs.

#### 2.1.2. In Situ Decoration of MWCNTs with Ag Nanoparticles

Briefly, 5 wt% silver nitrate (AgNO_3_, CHIMREACTIV, Bucharest, Romania) was mixed for 10 min (in the dark) with 20 mL *N,N*-Dimethylformamide (DMF, EMSURE, Darmstadt, Germany), and then the obtained mixture was added dropwise in a solution of MWCNTs (5 wt%) dispersed in 10 mL distillated water. The resulting solution was sonicated for 1 h at room temperature, in the dark. Subsequently, a solution of 25 mL sodium borohydride (NaBH_4_, Scharlau, Barcelona, Spain) was added dropwise in the solution obtained above, in an ice-bath, and the solution was left without stirring for 1 h (for Ag deposition). Further, the final product was separated by centrifugation, washed with distillated water several times and dried at 80 °C for 8 h.

### 2.2. Characterization Methods

#### 2.2.1. Fourier Transformed Infrared Spectroscopy (ATR–FTIR):

ATR-FTIR was recorded with a GX-type FTIR spectrometer (Perkin Elmer, Waltham, MA, USA), in the 4000–400 cm^−1^ range, with a resolution of 4 cm^−1^, by accumulating and mediating 32 spectra. This technique is very useful to determine the purity of the synthetized materials [28].

#### 2.2.2. Raman Spectroscopy Analysis

Raman spectroscopy was performed using a Horiba equipment (Labram HR Evolution, Pailaiseau, France) with an excitation wavelength of 514 nm and a 50× objective with a 10 s acquisition time. This technique is one of the most important tools for CNTs characterization, because it is a nondestructive analysis and is performed without sample preparation [28].

#### 2.2.3. X-ray Diffraction Analysis (XRD)

XRD was carried out with a Rigaku Ultima IV diffractometer (Rigaku, Tokyo, Japan) using Cu Kα radiation (λ = 1.54 Å). In this experiment, the accelerating voltage of the generator radiation was set at 40 kV and the emission current at 200 mA. The diffractograms were recorded in parallel beam geometry over 2θ = 10° to 90° continuously at a scan rate of 4°/min. This technique is used to obtain information on the interlayer spacing, the structural strain and impurities [28].

#### 2.2.4. Transmission Electron Microscopy (TEM)

TEM was achieved using a G2 F20 TWIN Cryo-TEM (Philips, Eindhoven, Netherlands) with an accelerating voltage of 200 keV. The samples were previously dispersed in distillated water and sonicated for one hour. One drop of the aqueous dispersion was placed on the holey formvar grid and microscopically examined. TEM is an essential tool for characterizing the nanomaterials because it provides information about the material’s shape and structure [28].

#### 2.2.5. Environmental Scanning Electron Microscopy with Energy-Dispersive X-ray Analysis (ESEM/EDX)

ESEM/EDX analysis was achieved using an FEI-Quanta 200 microscope incorporated with EDX spectrum profiling (ESEM-FEI Quanta 200, Eindhoven, Netherlands) and imaging was used in order to investigate the morphology of the resulting nanomaterials. ESEM images were obtained in a low vacuum mode, without any covering of the samples. This technique is used to obtain information about surface topography and composition [28].

#### 2.2.6. Brunauer–Emmett–Teller (BET) Analysis

BET surface area analysis was used for porosity determination, and the nitrogen adsorption/desorption isotherms were recorded at 77 K in the relative pressure range p/p_0_ = 0.005–1.0, using a NOVA2200e Gas Sorption Analyzer (Quantachrome, Boynton Beach, FL, USA). NovaWin version 11.03 software was used for data processing. Prior to adsorption measurements, the samples were degassed for 4 h at 180 °C under vacuum. The specific surface area was determined by the standard Brunauer–Emmett–Teller equation. The total pore volume was estimated from the volume adsorbed at a relative pressure p/p_0_ close to unity. Pore size distribution and pore volume were obtained from the desorption branch of the isotherm by applying the Barrett–Joyner–Halenda (BJH) model.

#### 2.2.7. Absorbance Variation in Time

The absorbance was determined with a JKI Spectrophotometer 721 N (Shanghai Jingke Scientific Instrument Co., Ltd., Shanghai, China). The absorption was recorded at 330 nm and the samples were measured during different time intervals between 0 and 5 h of incubation. The mixtures were stored at room temperature in quartz cuvettes in order to investigate their dispersion in aqueous media in time, an important feature of the nanomaterials to be used in various applications. Three measurements were investigated for each sample, at specified times, and the results were reproducible.

### 2.3. Antimicrobial Analysis

#### 2.3.1. Microbial Strains and Growth Conditions

*S. aureus* ATCC 25923, *B. subtilis* ATCC 6633, *P. aeruginosa* ATCC 25324, *E. coli* ATCC 25922 and *C. albicans* ATCC 10231, purchased from American Type Culture Collection (ATCC, Manassas, VA, USA), were used for the antimicrobial analysis. Glycerol stocks were streaked on LB agar (for bacteria) or Sabouraud agar (for *C. albicans*) to obtain fresh cultures in order to be used for subsequent studies. These strains were chosen due to the difference in terms of internal structure and cellular wall composition. Since numerous studies investigating nanostructured materials are reporting that antimicrobial mechanisms are correlated with cellular wall, a difference in the antimicrobial efficiency of the studied materials against the investigated pathogens can be expected.

#### 2.3.2. Qualitative Antimicrobial Assay

An adapted method of disk diffusion was used to evaluate the qualitative antimicrobial effects of the nanomaterials tested. Microbial suspensions were obtained for each strain and adjusted to an optical density of 0.5 McFarland (1.5 × 10^8^ CFU/mL). The suspensions were used to swab inoculate the entire surface of the nutrient agar into Petri dishes. After inoculation, 6 mm sterile absorbent paper discs were placed on the surface of the inoculated agar. Fresh solutions dispersed in distilled water were obtained and adjusted to 5 mg/mL for each of the obtained materials. Each solution of known concentration was placed on a separate sterile disk in a volume of 5 μL and the Petri dishes were incubated for 24 h at 37 °C to allow microbial growth. After incubation, the diameter of the growth inhibition zone (mm) was measured.

#### 2.3.3. Minimum Inhibitory Concentration (MIC) Assay

In order to investigate the MIC value of the compounds, a quantitative method was used, in which microdilutions were performed in liquid medium (nutritive “broth” Luria for bacteria and Yeast Peptone Glucose broth for *C. albicans*). Sterile broth was added to 96-well sterile plates and binary dilutions of each test compound were made in a volume of 150 μL (eight dilutions were investigated, respectively: 1, 0.5, 0.25, 0.125, 0.0625, 0.03125, 0.0156 and 0.0078 mg/mL of compounds, starting from an initial concentration of 1000 µg/mL). After microdilutions, 15 μL of 0.5 McFarland density microbial suspension was added to each well. The seeded plates were incubated for 24 h at 37 °C, and after incubation, the MIC value for each compound was determined by visual analysis and confirmed spectrophotometrically (absorbance at 600 nm). Each experiment was performed in triplicate and repeated on at least three separate occasions.

#### 2.3.4. Biofilm Development

In order to investigate the effect of the obtained materials on the adhesion and production of biofilms, a quantitative method was used, based on the realization of binary microdilutions in liquid medium, distributed sterile in 96-well plates, in a final volume of 150 μL. Subsequently, with a micropipette, eight binary dilutions were made, starting from well 1 (concentration of 1 mg/mL), to well 8 (where the final concentration was 0.0078125 mg/mL). After microdilution, 15 μL of 0.5 McFarland density microbial suspension (1.5 × 10^8^ CFU/mL) was added to each well. The seeded plates were incubated for 24 h at 37 °C. After incubation, the formed biofilms were carefully washed 3 times with sterile phosphate buffer saline (PBS) and fixed with cold methanol for 5 min. After removing the methanol, the dry plates were stained with 1.5% violet crystal solution for 20 min. After staining, the excess dye was washed with tap water, and the dye included in the cells of the biofilm formed on the walls was solubilized with a 33% acetic acid solution. The suspensions thus obtained were used for the interpretation of the results, based on the spectrophotometric reading of the absorbance of the colored suspension at 492 nm. Each experiment was performed in triplicate and repeated on at least three separate occasions.

#### 2.3.5. Statistical Analysis

The data were entered into Microsoft Excel 2013 and analyzed by using data analysis ad-ins. Single-factor and two-factor ANOVA was applied and *p*-value < 0.05 was considered statistically significant. All the experiments were repeated for triplicate values.

## 3. Results and Discussions

The research is focused on investigating the proper in situ decoration of MWCNTs with NPs, their dispersion in water and their antimicrobial properties.

### 3.1. FTIR Spectroscopy

**Figure 1:**
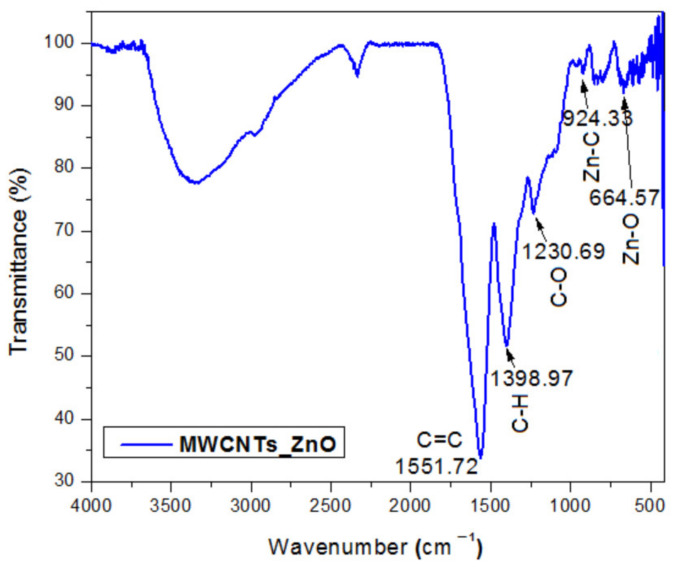
FTIR spectra of the MWCNTs_ZnO.

The infrared spectrum of MWCNTs_ZnO is presented in Figure 1. The FTIR spectra shows bands at 1398 and 1551 cm^−1^ that are related to the C–H and C=C bonds characteristic of carbon nanotubes’ skeleton. The peaks from 1230 cm^−1^ (C–O bonds) and 3500 cm^−1^ (–OH groups) appear due to possible leads of water and acids used during the purification process, identified from the base sample (MWCNTs) and reported in our previous study [18]. The peaks at 664 and 924 cm^−1^ are related to the characteristic stretching mode of Zn–O and Zn–C bonds from the MWCNTs_ZnO nanocomposite [29]. According to these results, the successful synthesis of MWCNTs_ZnO nanosystems was demonstrated.

**Figure 2:**
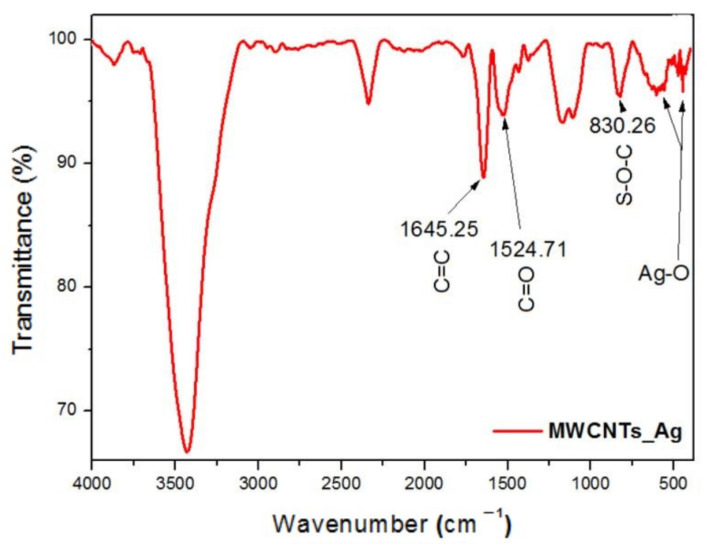
FTIR spectra of the MWCNTs_Ag.

The infrared spectrum of MWCNTs_Ag is presented in Figure 2. The FTIR spectra shows an intense peak centered at 3425 cm^−1^ related to the –OH groups, and the sharp peak at 1640 cm^−1^ that corresponds to C=C bonding of aromatic rings of the carbon skeleton structure. The peaks at 1524 (C=O), 1171 and 1100 cm^−1^ (CNT–COOH), and at 830 cm^−1^ (S–O–C), are attributed to the efficient functionalization of nanotubes using the aggressive oxidation treatment with HNO_3_/H_2_SO_4_ mixture [21,22,30]. Additionally, the small peaks between 613 and 481 cm^−1^ correspond to the deformation vibrations of the metal oxygen bond between Ag–O [22], proving the MWCNTs decoration with Ag NPs. The successful synthesis of MWCNTs_HAp has been reported in our previous study [18].

### 3.2. Raman Analysis

**Figure 3:**
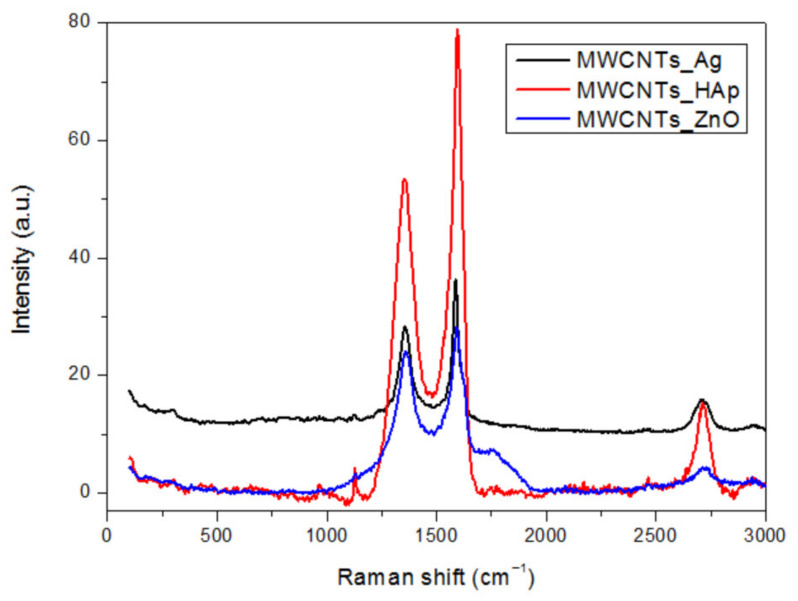
Raman spectra of MWCNTs decorated with NPs.

Raman spectra of MWCNTs_HAp, MWCNTs_ZnO and MWCNTs_Ag are presented in Figure 3. Three bands corresponding to the crystalline carbon D (band D—disorder), G band (graphite band) and G′ band (D overtone) characteristic of MWCNTs were observed at 1348, 1606 and 2698 cm^−1^, respectively. The D band indicates the disordered or hybridized carbon atoms from the wall of the nanocomposites (Table 1). The D band characteristic to MWCNTs is shifted after decoration at higher wavenumbers, proving the appearance of small structural defects on nanotubes’ surface induced by the physical or chemical interaction between the NPs and MWCNTs. It can be observed that MWCNTs_ZnO presented the higher displacement, due to the structural defects caused in the carbon wall by the formation of a nanoparticle–nanotube coordination bond. Additionally, smaller sizes of Zn NPs were observed, that can penetrate the nanotube structure more easily and can lead to a higher D shift. The G’ peak of the decorated MWCNTs shifted to a higher wavenumber, compared with the pure MWCNTs, suggesting both a substantial change transfer interaction between the NPs and nanotubes and a decrease in mass fraction of MWCNTs in the nanomaterials [22]. A smaller I_D_/I_G_ band ratio indicates a greater graphitic crystallinity of the MWCNTs. The increasing of the I_D_/I_G_ band ratio occurs due to the structural defects in the carbon wall, and this fact confirms the modification of the outer layers of the MWCNTs. Overall, the Raman spectrum confirms the decoration of nanotubes with NPs and indicates a good crystallinity with a change in maximum intensities [31].

### 3.3. XRD Analysis

**Figure 4:**
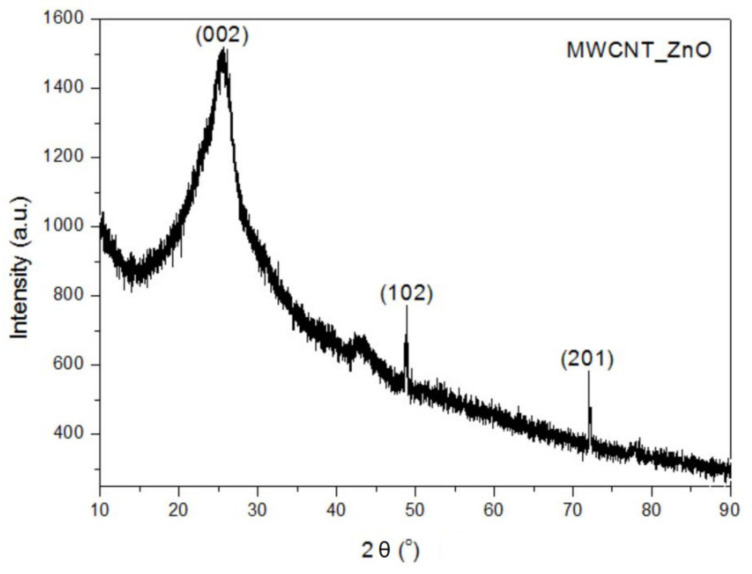
XRD of MWCNTs_ZnO.

**Figure 5:**
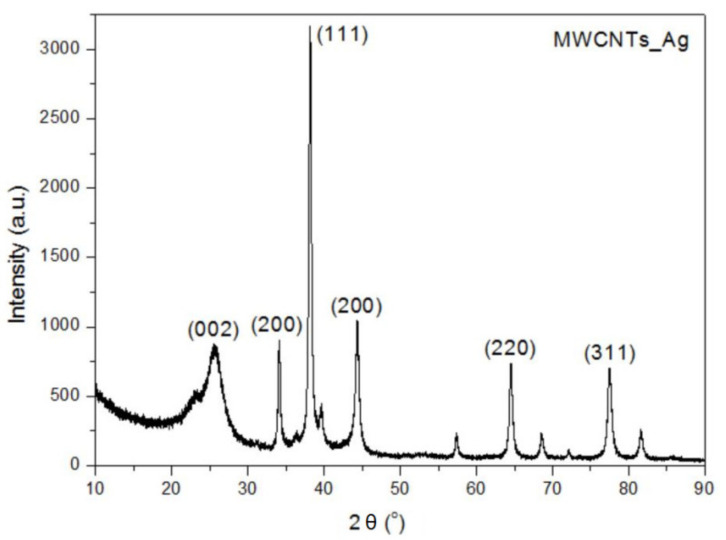
XRD of MWCNTs_Ag.

The XRD pattern of the MWCNTs_ZnO reveals the presence of the diffraction peaks corresponding to MWCNTs structure (25.53° and 42.86°). The characteristic diffraction peaks of ZnO nanostructure were observed at 48.59° and 71.67°, corresponding to (102) and (201) crystal planes of ZnO (Figure 4) [32,33]. In the case of MWCNTs_Ag, the sharp diffraction peaks are related to the Ag NPs, such as the distinct peaks of the 2θ values of 34.12°, 38.13°, 44.21°, 64.37° and 77.54° (Figure 5) [34,35,36].

The crystallite size (*L*) of the MWCNTs was calculated by using the Scherrer’s Formula (1) and the crystallite size for each nanomaterial is presented in Table 2.

(1) L=Kλ/βcosθ

where, *K* is Scherrer constant (0.91), *λ* is the X-ray wavelength (1.5406 Å), *β* is the full-width at half maximum and *θ* is the Bragg angle (rad).

XRD confirmed the phase purity of the synthesized nanomaterials as no additional phases were identified. In the case of MWCNTs_HAp [18] and MWCNTs_Ag, the crystallite size significantly increases due to the higher size of NPs that covered the surface of the nanotubes. In addition, these samples show the lowest β distribution, resulting in the largest crystallite size and lowest lattice strains [37]. The lower crystallite size achieved for MWCNTs_ZnO occurs due to the higher temperature to which the material is subjected in the synthesis method. The temperature and pressure influence the crystalline structures: at high temperatures, impurities or trapped gases come out and atoms, which are not at the minimum energy level, try to resettle, leading to a lower crystallite size. Therefore, higher temperature of calcination not only lowers the particle size, but also improves the crystallinity [38].

### 3.4. TEM Analysis

**Figure 6:**
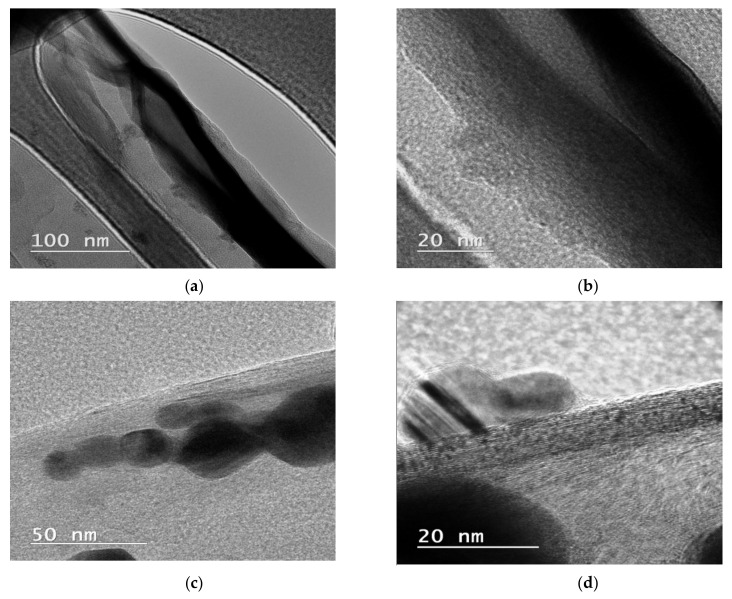
TEM micrographs of the MWCNTs_ZnO (**a**,**b**) and MWCNTs_Ag (**c**,**d**).

MWCNTs obtained in our previous work [18] presented a wide size distribution, having a diameter between 9 and 50 nm and a length of approximately 600 nm, with few defects, which suggests that nanotubes are composed of quality graphite layers. In Figure 6, the nanoparticles are deposited on the nanotubes’ surface, revealing the contact between the MWCNTS and NPs, confirming, thus, the successful decoration of the tubes. In the case of MWCNTs_ZnO, spherical NPs were observed on the nanotubes’ surface. The NPs appear to have a narrow size distribution, with dimensions between 7.41 and 13 nm. In the case of MWCNTs_Ag, the deposited NPs found on the nanotubes’ surface presented a diameter between 15.18 and 33.68 nm. However, the low-magnification image (Figure 6b,d) shows a homogeneous sample, with individual nanotubes covered with slightly aggregated nanoparticles. For MWCNTs_HAp, the attached NPs on the MWCNTs surface have diameters of about 35 nm, as reported from our previous study [18].

### 3.5. ESEM/EDX Analysis

**Figure 7:**
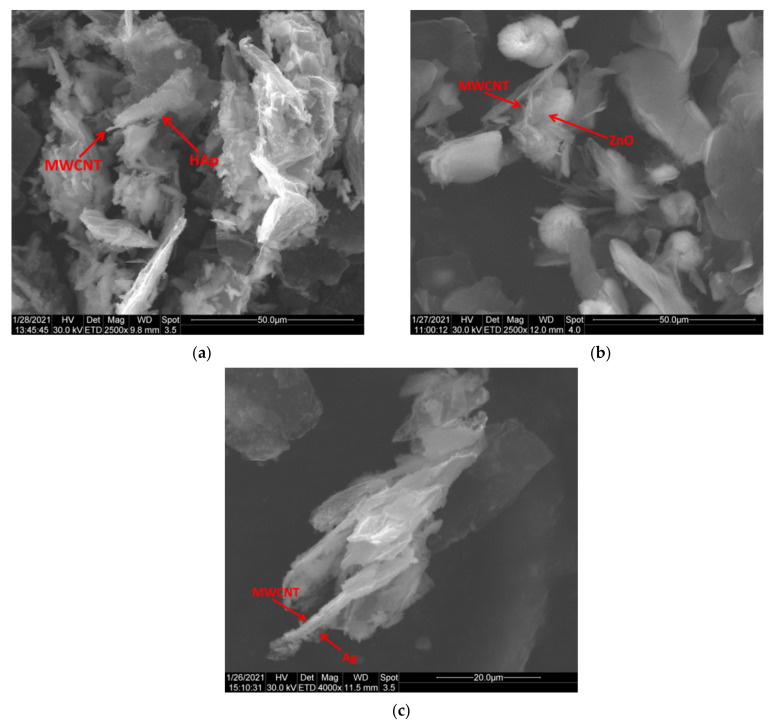
SEM images illustrating the decoration of MWCNTs with HAp (**a**), ZnO (**b**) and Ag (**c**) NPs.

The ESEM results sustain the successful decoration of MWCNTs with HAp, ZnO and Ag NPs, as previously demonstrated. It can be observed that the NPs have been deposited and cover the surface of MWCNTs to a large extent (Figure 7). After the nanotubes’ purification, that implies acid treatment, it is possible that the MWCNTs structure may be slightly modified and some bundles can be exfoliated and curled [39]. However, the results obtained by TEM confirm the fact that the structure of MWCNTs remains basically intact after decoration. In all the cases, the NPs are attached to the surface of MWCNTs and the formed exterior layer covers the MWCNTs evenly, mostly in the case of MWCNTs_Ag. In Figure 7a, the exterior layer of rod-like HAp NPs partially covers the nanotubes, and in some areas, agglomerations of particles are observed. In Figure 7b, the ZnO NPs did not completely cover the surface of nanotubes, and agglomerates with varying irregular shapes were observed on portions of the nanotube surface, while in the case of MWCNTs_Ag (Figure 7c), the entire surface of MWCNTs was uniformly covered by nanoparticles. The agglomeration of the NPs indicates the supporting role of the MWCNTs, as a center for the deposition of the clusters [40].

Decoration of MWCNTs with NPs was confirmed by the elemental quantitative analysis achieved by EDX and the percent of all elements in the different composites is listed in Table 3. The only presence element in MWCNTs is carbon and oxygen connected with the purification process of the MWCNTs. In addition, EDX analysis confirms the presence of Zn, Ca, P or Ag for each characteristic sample, that are in good agreement with their stoichiometric composition.

### 3.6. Brunauer–Emmett–Teller (BET) Surface Area Analysis

Table 4 presents the textural properties of the investigated MWCNTs and decorated MWCNTs. It can be observed that the MWCNTs_HAp and MWCNTs_ZnO are characterized by a larger surface area, due to the presence of the nanoparticles found on the MWCNTs surface. This could be directly related to the higher mean pore diameter of the NPs that decorate the surface of the nanotubes, compared with MWCNTs. Additionally, the mean pore diameter could be related with the dimension of particles found on the surface of MWCNTs. In the case of MWCNTs_Ag, the surface area decreased due to the fact that the NPs uniformly covered the entire surface of the nanotubes, and also because of the low surface area of Ag NPs (approximately 24 m^2^/g [41]), as compared with the nanotubes’ surface area.

**Figure 8:**
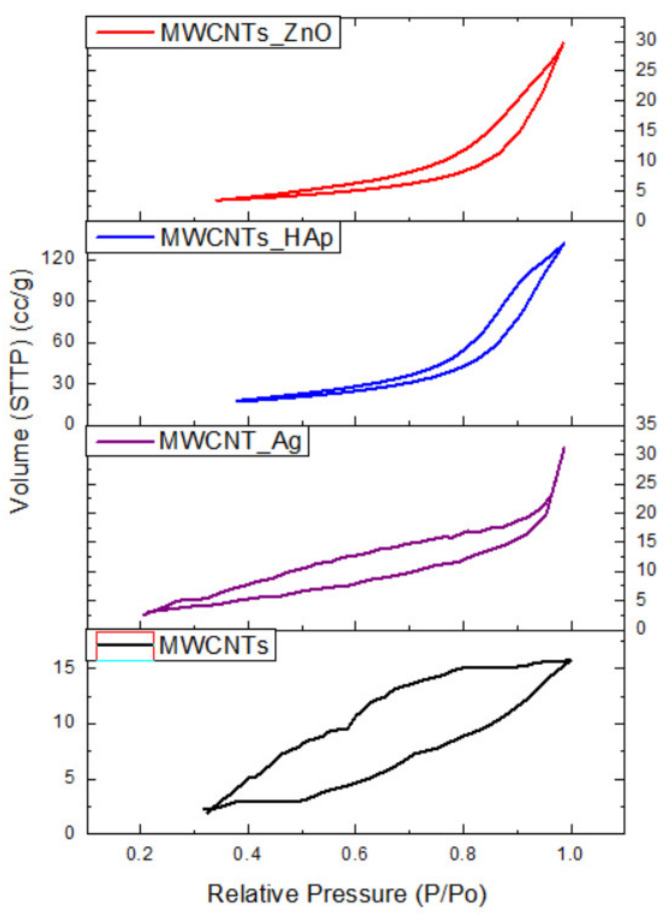
Isotherm curves of the MWCNTs and decorated MWCNTs.

Isotherm absorption/desorption curves for the MWCNTs and MWCNTs decorated with NPs are presented in Figure 8. The absorption/desorption isotherm of a nonreactive gas, at the temperature of 180 °C with 4 h heat treatment, is determined as a function of the relative pressure. At a minimum pressure, the smallest pores are filled with liquid nitrogen, as the pressure increases, larger pores are filled, and near the saturation pressure, all the pores are filled. The desorption process occurs when the pressure decreases form the saturation pressure [42]. The MWCNTs exhibited a type IV like isotherm with a H2-hysteresis loop, indicative that the distribution of pore size is not well-defined, according to the IUPAC classification [43]. After the decoration process, the shape of the hysteresis loop changed to a type V-like isotherm with a H3-hysteresis loop, due to the presence of NPs on the MWCNTs surface. This change suggests the presence of non-rigid aggregates of plate-like particles giving rise to slit-shaped pores, according to the IUPAC classification [43].

### 3.7. Variation in Time of Relative Absorbance

Dispersion is a process where agglomerated particles are separated from each other and a new interface between an inner surface of the liquid dispersion medium and the surface of the particles to be dispersed is generated. A system is stable when its dispersed phase can exist as separate individual particles for a long time [44]. Carbon nanotubes present structures with very complex morphologies, such as high number of van der Waals interactions that cause extremely poor dispersion in water or organic solvents. The nanotubes’ insolubility has become an important limitation for the practical applications of this unique material. In this study, the aqueous dispersion of MWCNTs and decorated MWCNTs with NPs has been investigated, because the proper dispersion of the nanomaterials is an important characteristic in order to retain their properties. UV-vis analysis is used to understand the suspension stability and aggregation by carefully observing the changes in the peak intensity, as well as spectral shape in the absorption spectra.

In this study, 1.5 mg of MWCNTs and decorated MWCNTs were dispersed in 2 mL of water in a quartz cuvette for 15 min under an ultrasonic bath in water, in order to investigate their dispersion.

**Figure 9:**
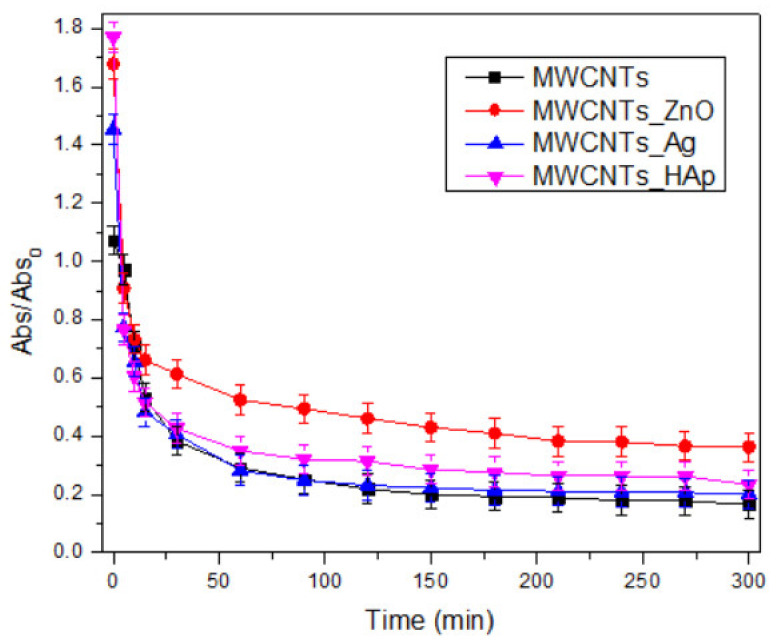
Variation in time of relative absorbance for MWCNT-based solution dispersed in water. Mean values of triplicate independent experiments and standard deviation are shown.

In Figure 9, mean values of relative absorbance vs. time were represented for each sample. It can be observed that compared to MWCNTs, all the decorated nanotubes presented a better dispersion in water. The slight increase of the dispersion of the MWCNTs obtained in our previous study [18], compared with other data from the literature [45,46], is due to the fact that the nanotubes used in this study were purified using oxidizing acid mixtures (sulfuric and nitric acids) that impart functional groups (–OH, −COOH) to nanotubes’ surface. The hydrophilic groups improve the dispersion in water of decorated MWCNTs.

Additionally, the nanotubes decorated with ZnO NPs presented the most increased dispersion in time because of the small size of the NPs attached to the MWCNTs surface, approximately 10 nm, while a larger particle size of approximately 30 nm is found on the surface of the MWCNTs_HAp and MWCNTs_Ag. Thus, the spectral changes suggest that smaller particles interact more with water molecules than larger particles [47,48].

### 3.8. Antimicrobial Analysis

#### 3.8.1. Qualitative Antimicrobial Assay

**Figure 10:**
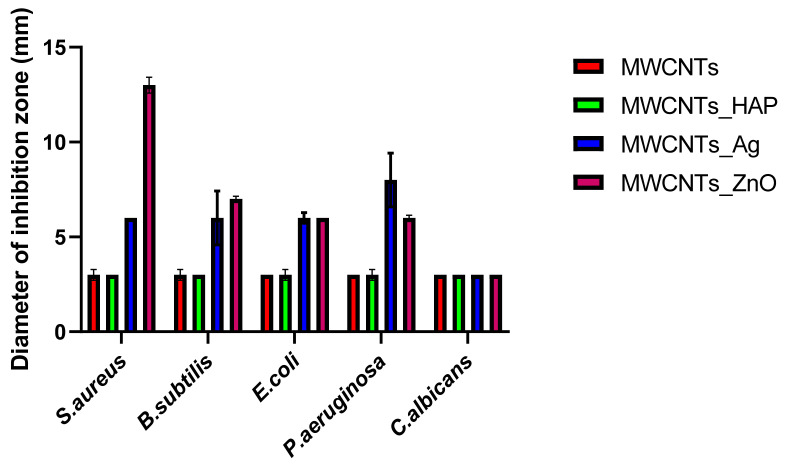
Graphical representation of the diameter of the growth inhibition zone of the microbial strains tested for MWCNTs and decorated MWCNTs. Data represented were mean of triplicate values with standard deviation.

Qualitative testing of antimicrobial activity demonstrated that MWCNT-based nanomaterials were able to inhibit growth for all tested strains. The length of CNTs is a very important aspect due to the interactions with cell membrane. The shorter tubes exhibit higher antimicrobial performance, compared with longer tubes. This fact is related to the interaction between nanotube and bacteria, leading to more cell membrane damage [39]. The nanotubes used in this study are short nanotubes (of about 600 nm length), this feature representing an advantage in improving the materials’ antimicrobial properties. However, the inhibition of bacterial growth was greatly improved with the nanoparticles’ decoration. The test showed that MWCNTs decorated with ZnO and Ag presented the largest area of inhibition for almost all tested strains, as compared with pure MWCNTs (Figure 10). The increased antimicrobial activity of MWCNTs_ZnO and MWCNTs_Ag occurs due to the presence of the NPs, because their action mode can disrupt bacterial cell membrane integrity, reduce cell surface hydrophobicity and downregulate the transcription of oxidative stress-resistance genes in bacteria [49,50,51,52]. In the case of MWCNTs_HAp, the antimicrobial activity was not improved compared with the activity of the nanotubes, because, in general, HAp does not show antibacterial properties [53]. For *C. albicans* strain, the areas of growth inhibition were minimal for all types of nanomaterials tested. These results suggest that the efficiency of the obtained materials is limited against yeasts. This can be related with particularities of their cellular wall (two-layered structure) and eukaryote organization.

#### 3.8.2. Minimum Inhibitory Concentration (MIC) Assay

**Figure 11:**
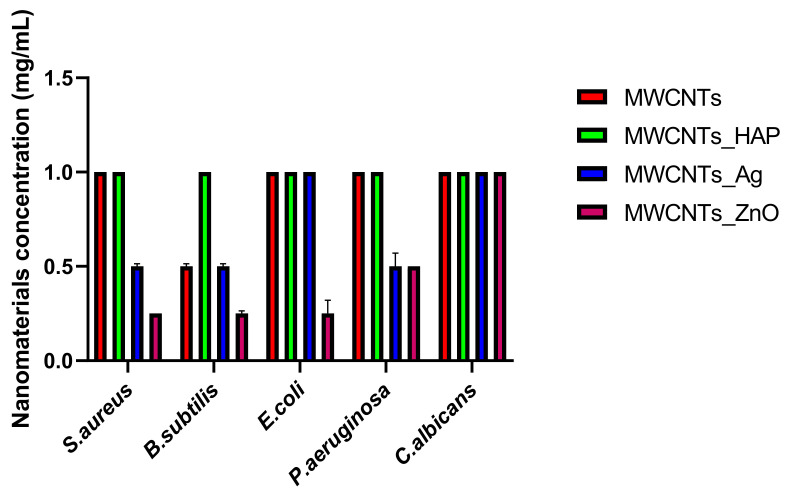
Graphical representation of MIC values of MWCNTs and decorated MWCNTs on microbial strains tested at 24 h incubation at 37 °C. Data represented were mean of triplicate values with standard deviation.

Quantitative tests showed that MWCNTs decorated with ZnO NPs have three times more pronounced antimicrobial effects compared to the pure nanotubes (Figure 11). This result can be correlated with the fact that ZnO NPs used to decorate nanotubes are able to enhance and prolong antibacterial activity [54,55]. Distinctive mechanisms of antibacterial activity of ZnO NPs have been reported in the literature, for example, the direct contact of ZnO NPs with cell wallsleads in destructing bacterial cell and ROS formation [56]. The values of the minimum inhibitory concentration were also influenced by the microbial strain tested, for example, in the case of MWCNTs_ZnO, the CMI values obtained for *S. aureus*, *B. subtilis* and *E. coli* were 0.25%, while the MIC value for *P. aeruginosa* was 0.5%. In addition, MWCNTs_Ag presented improved antimicrobial effects that confirmed that the presence of the attached NPs has a great importance; physical interactions between nanocomposites and cell membrane take place, which leads to a faster destructibility of the cell membrane. In the case of MWCNTs and MWCNTs_HAp, the MIC was 1 mg/mL for almost all strains tested, confirming a slightly lower antimicrobial activity.

#### 3.8.3. Biofilm Development

**Figure 12:**
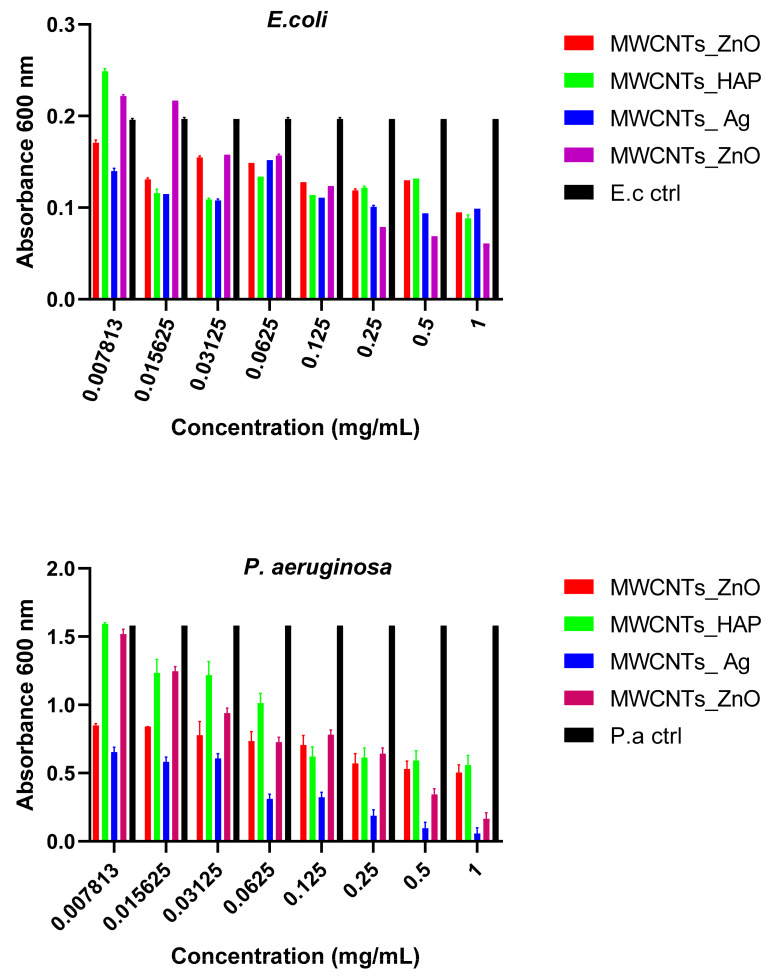
Graphical representation of the minimum concentration for eradication of biofilm for MWCNTs and decorated MWCNTs on Gram-negative strains. Data represented were mean of triplicate values with standard deviation.

**Figure 13:**
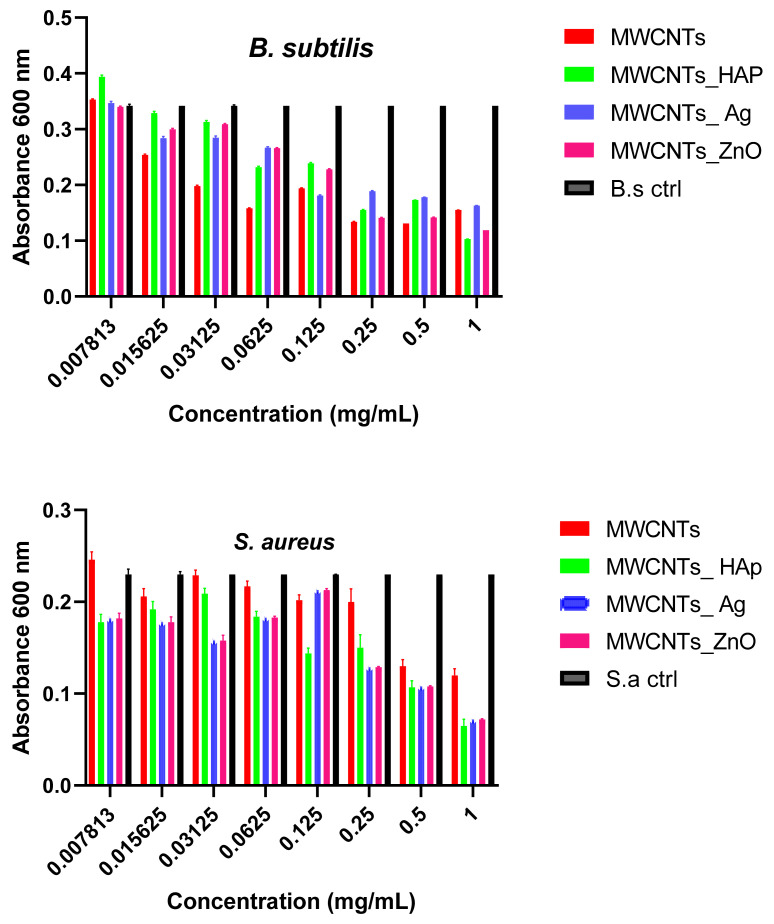
Graphical representation of the minimum concentration for eradication of biofilm for MWCNTs and decorated MWCNTs on Gram-positive strains. Data represented were mean of triplicate values with standard deviation.

**Figure 14:**
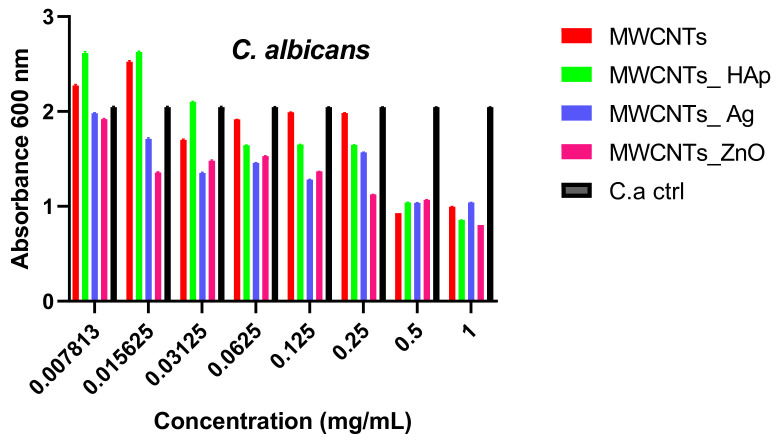
Graphical representation of the minimum concentration for eradication of biofilm for MWCNTs and decorated MWCNTs on the fungal pathogen *C. albicans*. Data represented were mean of triplicate values with standard deviation.

The biofilm formation test showed that the nanomaterials obtained reduce the ability of microorganisms to develop biofilms in a dose- and strain-dependent manner. Inhibition was observed at concentrations between 0.031% and 1%, for all strains, and the highest anti-biofilm effect was observed for *E. coli* and *P. aeruginosa* strains (Figure 12). This occurs due to the sensitivity of bacteria to NPs that varies according to the interface provided by the bacterial membrane. It was demonstrated that Gram-positive bacteria are less sensitive to NPs, compared to Gram-negative bacteria, due to the presence of a thicker peptidoglycan layer [57]. Therefore, different effects of the nanomaterials tested on different microbial strains were observed due to the variety and versatility of the microbes, which behave very differently under similar conditions (Figure 12, Figure 13 and Figure 14). Their distinct behavior can be explained by their bio-chemical peculiarities, the morphology of the cell wall or their ability to produce biofilms [58]. In the case of biofilm formation, the tendency is maintained, and MWCNTs and MWCNTs_HAp present a higher capacity of biofilm formation at the lowest concentrations.

*C. albicans* are eukaryotic cells and their internal structure and cellular wall composition (two-layered structure) is different, as compared with bacteria. In Figure 14, the graphical representation of the minimum concentration for eradication of biofilm for the obtained nanomaterials on the fungal pathogen *C. albicans*, is presented. It can be observed that, at higher concentrations, the materials are more efficient in the biofilm eradication. As the nanocomposites concentration decreases significantly (at 0.007% and 0.015%), their efficiency decreases and the yeast pathogen shows a high tolerance to these nanomaterials.

### 3.9. Data Analysis

The analysis of variance (ANOVA: single factor) for stability of water dispersion of MWCNTs, MWCNTs_ZnO, MWCNTs_HAp and MWCNTs_Ag by measuring the relative absorbance in time is presented in Table 5. This can be achieved by SS (sum of square) and *F*-value determination. According to these results, at the 5% level, the main factors (absorbance and time) have a reasonable effect on stability determination. No insignificant factors were obtained, thus, the stability of the MWCNTs and decorated MWCNTs can be measured by UV analysis. According to *p*-values, the decorated nanomaterials are more stable in time, compared with MWCNTs.

Table 6, Table 7, Table 8 and Table 9 list the results of statistical analysis of the biofilm eradication capacity. The prediction model consisted in the fact that the specified nanomaterials are efficient against *S. aureus*, *B. subtilis*, *P. aeruginosa*, *E. coli* and *C. albicans*. The importance of the main factors, such as nanomaterials’ concentration and absorbance for each strain after biofilm eradication, are considered for predicting the antimicrobial activity of MWCNTs, MWCNTs_ZnO, MWCNTs_HAp and MWCNTs_Ag against Gram-positive and Gram-negative strains and fungal pathogens. The degrees of freedom (df) for concentration and absorbance were 7 and 4, respectively. The SS can be applied to investigate the adequacy of the proposed models, with the smallest values being obtained for the MWCNTs_ZnO and MWCNTs_Ag. According to these results, at the 5% level of probability, the main factors, such as concentration of the nanocomposites and the type of strain used for analysis, have a reasonable effect on the biofilm eradication of the strains. Additionally, there were no insignificant factors and the SS and df of lack of fit were zero for all the nanocomposites. Based on the F-values of the main factors, the best antimicrobial activity was obtained for MWCNTs_ZnO and MWCNT_Ag. All the *p*-values, for all concentrations, strains’ absorbance and materials, were less than 0.05, so the proposed model was adequate, and the nanomaterials’ antimicrobial activity was highlighted.

## 4. Conclusions

In this study, we reported the decoration of MWCNTs with different types of nanoparticles, such as ZnO, Ag and HAp, in order to obtain hybrid materials with improved antimicrobial activity. The decoration of MWCNTs with NPs was confirmed by structural and morphological analyses, such as Fourier transformed infrared spectroscopy, Raman spectroscopy, X-ray diffraction, transmission electron microscopy, environmental scanning electron microscopy/energy-dispersive X-ray spectroscopy and Brunauer–Emmett–Teller surface area analysis. The nanoparticles attached on the MWCNTs surface presented variable diameters, from 7 to 13 nm for ZnO NPs, 15–33 nm for Ag NPs and about 35 nm for HAp. In all the cases, the NPs were attached and aggregated to the surface of MWCNTs, as demonstrated by SEM analysis. The agglomeration of the NPs indicates the supporting role of the MWCNTs, as a center for the deposition of the clusters. Additionally, the dispersion in aqueous medium of the obtained nanomaterials was investigated and all the decorated MWCNTs presented a better dispersion in water, demonstrating a proper dispersion of the nanomaterials that retain their properties. The in vitro results demonstrated that the obtained nanocomposites have a significant antimicrobial activity and reduce biofilm formation in many relevant microbial strains, such as *S. aureus*, *B. subtilis, P. aeruginosa*, *E. coli* and *C. albicans*. The higher antimicrobial activity was observed for MWCNTs_ZnO and MWCNTs_Ag, with the largest diameter of inhibition zone being obtained for these nanocomposites (13–6 mm for MWCNTs_ZnO and 6–7 mm for MWCNTs_Ag). The biofilm formation test demonstrated that MWCNTs_ZnO and MWCNTs_Ag presented the highest antimicrobial activity, with the inhibition occurring even at lower concentrations, such as 0.015% and 0.007%. These results were supported by the statistical analysis. The SS was applied in order to investigate the adequacy of the proposed models, with the smallest values being obtained for the MWCNTs_ZnO and MWCNTs_Ag. In addition, based on the F-values of the main factors, the best antimicrobial activity was obtained for MWCNTs_ZnO and MWCNT_Ag. Thus, the obtained materials could be considered as competitive candidates for the development of efficient antimicrobial systems to fight against resistant pathogens and biofilm infections.

## Figures and Tables

**Table 1:** Relative intensity of D, G and G’ bands and I_D_/I_G_ band ratio of MWCNTs decorated with NPs.

Nanomaterials	D Band (cm^−1^)	G Band (cm^−1^)	G’ Band (cm^−1^)	I_D_/I_G_
MWCNTs [18]	1348	1606	2698	0.839
MWCNTs_Ag	1357	1613	2717	0.841
MWCNTs_HAp	1360	1615	2723	0.842
MWCNTs_ZnO	1367	1618	2734	0.844

**Table 2:** Summary of the XRD characterization of MWCNTs and decorated MWCNTs.

Sample	2θ, °	2θ, rad	β, °	L, Å	L, nm
MWCNTs [18]	25.53	0.4456	4.08	20.18	2.018
MWCNTs_HAp	25.79	0.4501	1.28	64.33	6.433
MWCNTs_ZnO	25.58	0.4464	6.59	12.49	1.249
MWCNTs_Ag	25.66	0.4479	2.036	40.46	4.046

**Table 3:** Chemical composition (EDX) of MWCNTs decorated with NPs.

Element	MWCNTs_ZnO	MWCNTs_Ag	MWCNTs_HAp [18]
C	38.21 ± 0.28	70.35 ± 0.81	26.33 ± 0.28
O	40.88 ± 0.53	12.35 ± 0.12	20.80 ± 0.22
Zn	19.91 ± 1.02	-	-
Ag	-	16.14 ± 0.6	-
Ca	-	-	31.04 ± 0.3
P	-	-	21.42 ± 0.23
Other	1.00 ± 0.072	1.16 ± 0.085	0.41 ± 0.014
Total	100	100	100

**Table 4:** BET surface area analysis of MWCNTs and MWCNTs decorated with NPs.

Sample	BET Analysis	
	Surface Area (m^2^/g)	Mean Pore Diameter (nm)
MWCNTs	35.121 ± 0.63	3.614 ± 0.56
MWCNTs_HAp	69.497 ± 1.79	13.705 ± 0.88
MWCNTs_ZnO	35.965 ± 0.94	3.810 ± 0.37
MWCNTs_Ag	25.016 ± 0.25	2.959 ± 0.08

**Table 5:** ANOVA table for variation in time of relative absorbance for MWCNTs and decorated MWCNTs dispersed in water.

Source of Variation	SS *	df *	MS *	*F*-Value	*p*-Value	
MWCNTs	100136.81	1	100136.81	17.92	0.000253568	Significant
MWCNTs_ZnO	99793.98	1	99793.98	17.86	0.000258447	Significant
MWCNTs_Ag	100104.95	1	100104.95	17.91	0.000254021	Significant
MWCNTs_HAp	100003.28	1	100003.28	17.90	0.000255464	Significant

* Sum of squares (SS), degrees of freedom (df), mean square (MS).

**Table 6:** ANOVA table of the minimum concentration for eradication of biofilm for MWCNTs on the investigated strains.

Source of Variation	SS *	df *	MS *	*F*-Value	*p*-Value	
Model	13.50	11	3.31	39.31	<0.0001	Significant
Concentration	0.69	7	0.11	2.98	0.0011	Significant
Absorbance	12.81	4	3.20	54.63	<0.0001	Significant
Lack of fit	0	0	-	-	-	-
Error	1.40	24	0.05	-	-	-
Total	14.91	35	-	-	-	-

* Sum of squares (SS), degrees of freedom (df), mean square (MS).

**Table 7:** ANOVA table of the minimum concentration for eradication of biofilm for MWCNTs_ZnO on the investigated strains.

Source of Variation	SS *	df *	MS*	*F*-Value	*p*-Value	
Model	7.31	11	1.86	25.69	<0.0001	Significant
Concentration	0.66	7	0.11	4.96	0.0019	Significant
Absorbance	6.65	4	1.66	74.08	<0.0001	Significant
Lack of fit	0	0	-	-	-	-
Error	0.53	24	0.02	-	-	-
Total	7.85	35	-	-	-	-

* Sum of squares (SS), degrees of freedom (df), mean square (MS).

**Table 8:** ANOVA table of the minimum concentration for eradication of biofilm for MWCNTs_HAp on the investigated strains.

Source of Variation	SS *	df *	MS *	*F*-Value	*p*-Value	
Model	13.40	11	3.25	29.04	<0.0001	Significant
Concentration	1.20	7	0.20	3.03	0.02	Significant
Absorbance	12.20	4	3.05	46.25	<0.0001	Significant
Lack of fit	0	0	-	-	-	-
Error	1.58	24	0.06	-	-	-
Total	14.98	35	-	-	-	-

* Sum of squares (SS), degrees of freedom (df), mean square (MS).

**Table 9:** ANOVA table of the minimum concentration for eradication of biofilm for MWCNTs_Ag on the investigated strains.

Source of Variation	SS *	df *	MS *	*F*-Value	*p*-Value	
Model	7.93	11	2.00	42.47	<0.0001	Significant
Concentration	0.31	7	0.05	3.36	0.01	Significant
Absorbance	7.62	4	1.90	123.27	<0.0001	Significant
Lack of fit	0	0	-	-	-	-
Error	0.37	24	0.01	-	-	-
Total	8.30	35	-	-	-	-

* Sum of squares (SS), degrees of freedom (df), mean square (MS).

## Data Availability

Not applicable.
